# Cardiomyopathie du péripartum

**DOI:** 10.11604/pamj.2018.29.7.12236

**Published:** 2018-01-04

**Authors:** Jihad Drissi, Zakaria Idri, Jaouad Kouach, Driss Moussaoui, Mohamed Dehayni

**Affiliations:** 1 Service de Gynécologie-Obstétrique, Hôpital Militaire d’Instruction Mohammed V, Rabat, Maroc

**Keywords:** Cardiomyopathie, péripartum, insuffisance cardiaque, choc cardiogénique, Cardiomyopathy, peripartum, heart failure, cardiogenic shock

## Abstract

La cardiomyopathie du péri-partum (CMP-PP) ou syndrome de Meadows, est une cardiomyopathie dilatée survenue pendant ou au décours d'une grossesse, définie par une insuffisance cardiaque avec une fraction d'éjection systolique du ventricule gauche inférieure à 45%. Il s'agit d'une entité pathologique rare dont le mécanisme physiopathologique en cause reste mal élucidé. Sur le plan clinique il s'agit d'une insuffisance cardiaque inopinée d'installation rapide, d'évolution imprévisible avec risque de choc cardiogénique réfractaire justifiant une prise en charge en réanimation cardiovasculaire. La CMP-PP ne nécessite aucun traitement spécifique par rapport aux autres causes d'insuffisance cardiaque. Nous rapportons le cas d'une primigeste de 29 ans qui a consulté à 32 semaines d'aménorrhée dans un tableau d'insuffisance cardiaque congestive en rapport avec une cardiomyopathie du péri-partum. L'objectif de ce travail est de préciser les caractéristiques de cette cardiopathie, qui malgré son caractère exceptionnel ne doit pas être méconnu par l'obstétricien.

## Abstract (en)

Peripartum cardiomyopathy (PP-CMP) or Meadows’ syndrom, is a dilated cardiomyopathy which occurs during or following pregnancy. It is characterized by heart failure with a systolic left ventricle ejection fraction of less than 45%. It is rare and its pathophysiologic mechanism is poorly elucidated. From a clinical point of view, it is characterized by unespected heart failure with rapid onset of symptoms, unpredictable evolution and risk of refractory cardiogenic shock, justifying patient’s management in Cardiovascular Unit. PP-CMP requires no specific treatment compared to other causes of heart failure. We here report the case of a 29-year old primip at 32 weeks of amenorrhea presenting with congestive heart failure associated with peripartum cardiomyopathy. The aim of this study was to highlight the features of this heart disease which, despite its rarity, should not be disregarded by obstetricians.

## Introduction

La cardiomyopathie du péri-partum (CMP-PP) ou syndrome de Meadows est une cardiomyopathie dilatée survenue pendant ou au décours d'une grossesse, définie par une insuffisance cardiaque avec une fraction d'éjection systolique du ventricule gauche inférieure à 45% [1,2]. Il s'agit d'une entité pathologique rare dont le mécanisme physiopathologique reste mal élucidé. L'objectif de ce travail est de préciser les particularités de cette cardiopathie qui malgré son caractère exceptionnel ne doit pas être méconnue par l'obstétricien.

## Patient et observation

**Figure 1:**
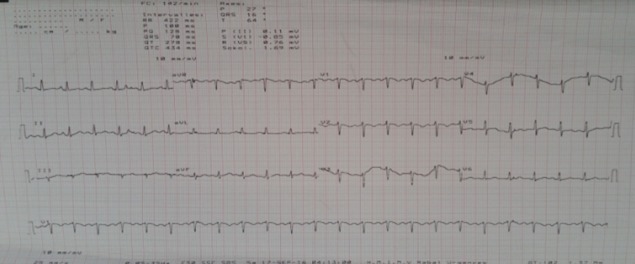
Tachycardie sinusale

**Figure 2:**
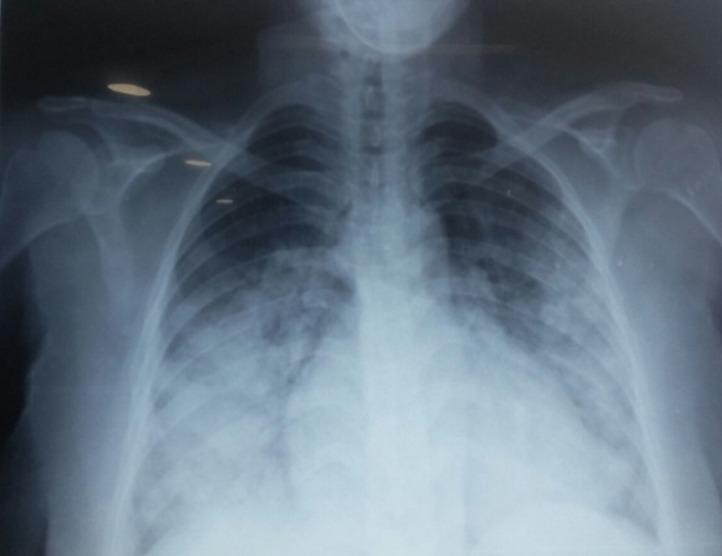
Radiographie pulmonaire de face: surcharge péri-hilaire en faveur d’un OAP

Nous rapportons le cas d'une primigeste de 29 ans, de groupe sanguin O+, sans antécédents pathologiques notables notamment pas d'antécédents personnels ou familiaux de cardiopathies, et sans antécédents de chimiothérapie ou de prise médicamenteuse, qui a consulté aux urgences à 32 semaines d'aménorrhée pour détresse respiratoire aigue avec une dyspnée stade IV de la NYHA, associée à une orthopnée sans douleurs thoraciques évoluant dans un contexte d'apyrexie. L'examen clinique a trouvé une patiente consciente, apyrétique, normotendue à 13/7cmHg, polypnéique, tachycarde à 110 battements par minute avec une désaturation SaO_2_: 55%, les mollets et cuisses souples. L'examen cardivasculaire a montré un assourdissement des bruits du cœur avec un bruit de galop, et des râles crépitants à l'auscultation pleuro-pulmonaire, sans signes d'insuffisance cardiaque droite. Par ailleurs, l'examen obstétrical, sans particularités, a trouvé une patiente en dehors du travail avec un score de Bishop défavorable. La radiographie pulmonaire a objectivé un syndrome alvéolo-interstitiel bilatéral en ailes de papillon, une silhouette cardiomédiastinale normale faisant évoquer un 35%, œdème aigu du poumon. L'électrocardiogramme a montré une tachycardie sinusale sans troubles de la repolarisation ventriculaire et sans signes d'ischémie myocardique. L'échographie cardiaque transthoracique a objectivé une fraction d'éjection ventriculaire gauche à 35%, sans dyskénésie segmentaire, sans dilatation cavitaire ni d'hypertrophie du myocarde et sans valvulopathies, avec un péricarde sec (Figure 1, Figure 2).

Le bilan biologique a objectivé un syndrome inflammatoire (CRP à 180 mg/l) et une élévation des enzymes cardiaques: troponine: 346ng/L, CPK: 222UI/L. Il s'agit donc d'un œdème aigu du poumon compliquant une insuffisance cardiaque lié à une myocardite virale ou à une cardiomyopathie du péri-partum. Devant la négativité des sérologies virales le second diagnostic est le plus probable. La patiente a donc été mise en position demi-assise, sous ventilation non invasive, diurétiques de l'anse et catécholamines. Avec cependant une aggravation clinique rapide; une désaturation profonde arrivant à 18% justifiant l'indication d'une extraction fœtale en urgence sous anesthésie générale avec intubation et ventillation assistée. La césarienne a permis l'extraction céphalique d'un nouveau-né de sexe masculin, poids de naissance à 2100g, apgar 6/10 passé à 9/10 qui a gardé une détresse respiratoire aigue cotée à 3/10 selon le score de Sylverman améliorée après administration de surfactant. L'évolution clinique maternelle était favorable avec une résolution de l'OAP sur les radiographies pulmonaires de contrôle, et une saturation normale à l'air ambiant après 24 heures d'intubation, elle était consciente, eupnéique et normotendue. Le contrôle échocardiographique a objectivé une amélioration de la fonction cardiaque avec une augmentation de la fraction d'éjection systolique ventriculaire gauche à 45%.

## Discussion

Décrite pour la première fois en 1997 par un groupe de travail du National Heart Blood and Lung Institute (NHBLI), la CMP PP se définit par une insuffisance cardiaque congestive avec une FEVG inférieure à 45% et/ ou une dilatation cavitaire avec un diamètre ventriculaire télédiastolique supérieur à 2,7cm/m^2^, survenant au cours du troisième trimestre de la grossesse ou les 5 mois suivant l'accouchement ( pic de fréquence dans les quelques jours du post-partum), sans cardiopathie préalable à la grossesse, avec un bilan étiologique complet demeuré négatif. En effet, la CMP-PP reste un diagnostic d'exclusion [1,2]. Entité pathologique rare qui représente moins de 1% des cardiopathies associées à la grossesse [3], son incidence est variable de 1/10000 à 1/4000 naissances. Plusieurs facteurs de risques ont été identifiés: âge supérieur à 30 ans, la multiparité, les grossesses multiples, la tocolyse prolongée, les carences vitaminiques et en oligoéléments notamment en sélénium. Cependant, un tiers des cas surviennent chez des patientes sans aucun facteur de risque [1,2]. Le mécanisme physiopathologique de la maladie est mal élucidé, plusieurs hypothèses sont évoquées: la théorie inflammatoire: proposée devant la constatation sur des biopsies myocardiques de lésions caractéristiques de myocardite aigue favorisées par les perturbations de la réponse inflammatoire(élévation des cytokines) associées à la grossesse déclenchées par un stress oxydatif induit entre autre par un agent infectieux. Dans ce sens, certains auteurs ont constatés une corrélation entre le taux d'anticorps anti-chlamydia [4] ou anti-viraux (VMV, coxakie B, parvovirus B19, EBV, HSV6…) et la survenu de la maladie [1,3]; l'hypothèse auto-immune: l'immunodépression relative de la grossesse permet de limiter d'éventuelles réactions contre les cellules foetales pouvant éventuellement passer dans la circulation maternelle. Cependant, si ces cellules foetales résident dans le tissu cardiaque après l'accouchement, le rétablissement d'une immunité maternelle normale pourrait être responsable d'une réaction contre ces cellules foetales, responsable de la dysfonction myocardique et de la CMP-PP. Ainsi, la présence d'anticorps anti-actine/myosine a été identifiée sur des biopsies myocardiques [5,6].

**La théorie hormonale:** par activation, suite à un stress oxydatif, de la cathepsine D (protéase) qui entraine un clivage intramyocardique de la prolactine en petites fractions de faible poids moléculaire (16KDa prolactine) à activité antiangiogénique et proapoptotique [6,7]. **La théorie génétique:** a été évoquée devant la constatation de formes familiales liées à des mutations de la troponine, des chaînes lourdes de la myosine ou des canaux sodiques. Il s´agirait donc probablement de cardiomyopathies latentes, révélées par la grossesse [6]. **La théorie métabolique par déficit en sélénium** a également été évoquée [7,8]. Le tableau clinique est celui d'une insuffisance cardiaque globale ou gauche d'installation rapide, voire, comme chez notre patiente, un œdème aigu du poumon inaugural. Un tableau de choc cardiogénique peut s'installer en quelques heures [1,2]. La radiographie pulmonaire montre une surcharge péri-hilaire en ailes de papillon en faveur d'un œdème aigu du poumon associée parfois à une cardiomégalie [1,2]. L'ECG: qui a objectivé une tachycardie sinusale chez notre patiente, trouve des signes non spécifiques à type de troubles du rythme ou de la repolarisation ventriculaire pouvant prêter confusion avec une éventuelle cardiopathie ischémique [1,2].

L'échocardiographie transthoracique, examen clé du diagnistic; confirme l'insuffisance cardiaque avec ou sans dilatation ventriculaire gauche et une possible atteinte cardiaque droite associée, une hypokinésie globale sans anomalies de la cinétique segmentaire [9]. L'examen recherche des complications à type de thrombus intra-cardiaque ou d'épanchement péricardique associé, et élimine certains diagnostics différentiels: une cardiopathie hypertrophique, une valvulopathie notamment rhumatismale ou une dyskinésie myocardique en faveur d'une cardiopathie ischémique. Le problème diagnostic différentiel se pose avec la cardiomyopathie dilatée primitive. Cependant, la dilatation importante des cavités cardiaques, l'amincissement pariétal marqué et un niveau important d'hypertention artérielle pulmonaire sont des éléments qui témoignent de l'ancienneté du trouble et donc de son caractère prégestationnel. Le diagnostic de myopéricardite peut être plus difficile à écarter devant un contexte d'infection virale ; l'IRM dans ce cas peut être d'une aide précieuse [5]. Le diagnostic d'embolie amniotique doit être éliminé afin de ne pas retarder une prise en charge spécifique et optimale [6,10]. Les explorations hémodynamiques invasives sont indiquées dans les formes gravissimes résistant au traitement médical pour lesquelles une assistance circulatoire extracorporelle sera discutée. Elle permet la réalisation simultanée d'une coronarographie afin d'éliminer une cause coronarienne en cas de présomption du diagnostic et permet la réalisation d'une biopsie endomyocardique: la constatation d'un aspect très inflammatoire ou d'un important infiltrat lymphocytaire peut faire poser l'indication d'un traitement immunosuppresseur, dont l'efficacité reste discutée [1,2].

Le traitement symptômatique vise à réduire la précharge, augmenter la contractilité myocardique et diminuer les résistances vasculaires périphériques [3]. Il repose, à l'instar des autres types d'insuffisance cardiaque, sur la limitation de l'activité physique, la restriction hydro-sodée, l'association de diurétiques de l'anse et de dérivés nitrés en cas de signes congestifs, à utiliser avec parcimonie vu le risque de déshydratation. La digoxine a un effet antiarythmique et ionotrope positif.Les bétabloquants peuvent être utilisés à faible doses en prépartum [11]. Les IEC seront réservés au post-partum en raison de leur néphrotoxicité fœtale. L'héparinothérapie s'impose vu le risque thromboembolique associé [1,2]. L'évolution de la maladie est totalement imprévisible, elle peut se faire en 24-48h vers un état de choc cardiogénique réfractaire nécessitant une assistance circulatoire extra-corporelle dans l'attente de la récupération fonctionnelle ou de la transplantation cardiaque [1,2]. Se référant à la théorie hormonale, la bromocriptine a été récemment proposée, elle sera contre-indiquée en cas d'antécédents d'accident thromboembolique artériel, en cas de thrombus intra-cardiaque ou en cas d'HTA sévère mal contrôlée ou de préeclampsie [1,2]. En cas de CMP-PP survenant en fin de grossesse l'extraction f'tale est souvent indiquée, elle se fera par voie basse si l'état du col le permet et la dyspnée est légère (stade I de la NYHA), l'anesthésie péridurale est indiquée dans ce cas car elle réduit le travail cardiaque et améliore la fonction cardiaque par diminution des conditions de charge, l'extraction instrumentale doit être facile [7]. Dans le cas contraire la césarienne est indiquée. La rachianesthésie seule est fortement déconseillée à cause des modifications hémodynamiques brutales qu'elle engendre et qui peuvent être fatales dans ce contexte. L'anesthésie péridurale ainsi que la rachianesthésie combinée à la péridurale (RCP) sont à préférer. L'anesthésie locorégionale est à privilégier sauf dans les situations d'urgence obstétricales et de contre-indication où l'anesthésie générale garde sa place [12].

Dans le cas d'une cardiopathie survenant avant terme il n'y a pas de recommandations systématiques, le risque de prématurité sera discuté au cas par cas en fonction de l'importance de la défaillance cardiaque, de l'évolution initiale sous traitement, de la survenue d'une souffrance fœtale [5]. L'évolution initiale en phase aigue est imprévisible avec risque d'état de choc cardiogénique réfractaire et de complications thromboemboliques. Le taux de mortalité est estimé à 10-15%. La mortalité péri-natale se situe aux alentours de 10% [13]. L'évolution à long terme est aussi imprédictible: une restitution ad integrum de la fonction cardiaque est observée dans la moitié des cas, dans un tiers des cas on note une stabilisation des lésions, seul un faible nombre va évoluer progressivement vers l'insuffisance cardiaque réfractaire indication d'une transplantation cardiaque. L'absence de récupération d'une fonction cardiaque intégrale dans les six mois du post-partum témoigne d'une évolution vers la cardiomyopathie chronique [1,4]. Se référant à la théorie hormonale l'allaitement maternel serait déconseillé. L'usage d'estrogènes est contre-indiqué vu le risque thromboembolique [7]. Le risque de récidive au cours d'une grossesse ultérieure est variable selon les études (25 à 100%), il semble plus élevé en cas d'insuffisance cardiaque séquellaire. Ainsi, la Société européenne de cardiologie préconise de déconseiller toute grossesse en cas d'insuffisance cardiaque persistante, de la contre-indiquer si la FEVG reste inférieure à 50% en raison du risque de décompensation cardiaque, de réaliser une échocardiographie de dépistage au troisième trimestre chez les patientes aux antécédents de CMP-PP complètement récupérée ou en cas d'antécédent familial de CMP-PP chez une apparentée de premier degré. Chez ces patientes la bromocriptine sera discutée au cours du dernier mois de grossesse car elle préviendrait la récidive [6].

## Conclusion

La CMP-PP est une entité pathologique, quoique rare, ne doit pas être méconnue par l'obstétricien. Elle sera évoquée devant toute insuffisance cardiaque congestive se manifestant en fin de grossesse ou en post-partum après avoir exclue les autres diagnostics étiologiques. L'évolution en phase aigue est imprévisible parfois fatale avec risque d'état de choc cardiogénique réfractaire. La prise ne charge est essentiellement symptômatique identique à toute défaillance cardiaque. Les IEC constituent le traitement de référence, mais ils restent contre-indiqués pendant la grossesse. D'autres thérapeutiques sont en cours d'évaluation et fonction des hypothèses étiologiques. La mortalité maternelle reste élevée. La normalisation secondaire de la fonction ventriculaire n'exclue pas un risque de récidive lors d'une grossesse ultérieure qui justifiera un suivi pluridisciplinaire.

## Conflits d’intérêts

Les auteurs ne déclarent aucun conflit d’'intérêts.
